# Anthropometrics, Metabolic Syndrome, and Mortality Hazard

**DOI:** 10.1155/2018/9241904

**Published:** 2018-07-12

**Authors:** Nir Y. Krakauer, Jesse C. Krakauer

**Affiliations:** ^1^Department of Civil Engineering, The City College of New York, New York, NY, USA; ^2^Metro Detroit Diabetes and Endocrinology, Southfield, MI, USA

## Abstract

Independent indices (height, body mass index, a body shape index, and hip index) derived from basic anthropometrics have been found to be powerful predictors of mortality hazard, especially when the attributable risks are summed over these indices to give an anthropometric risk index (ARI). The metabolic syndrome (MS) is defined based on the co-occurrence of anthropometric, clinical, and laboratory criteria and is also widely employed for evaluating disease risk. Here, we investigate correlations between ARI and MS in a general population sample, the United States Third National Health and Nutrition Examination Survey. Baseline values of ARI and MS were also evaluated for their association with mortality over approximately 20 years of follow-up. ARI was found to be positively correlated with each component of MS, suggesting connections between the two entities as measures of cardiometabolic risk. ARI and MS were both significant predictors of mortality hazard. Although the association of ARI with mortality hazard was stronger than that of MS, a combined model with both ARI and MS score as predictors improved predictive ability over either construct in isolation. We conclude that the combination of anthropometrics and clinical and laboratory measurements holds the potential to increase the effectiveness of risk assessment compared to using either anthropometrics or the current components of MS alone.

## 1. Introduction

In 2009, an international task force recognized metabolic syndrome (MS) as a critical component in addressing cardiovascular and mortality risk around the globe [1]. The diagnosis of MS is based on co-occurrence of hypertension (a biophysical clinical measurement); elevated triglycerides, low high-density cholesterol (HDL), and hyperglycemia (laboratory measures); and elevated waist circumference (WC, an anthropometric measurement). Though there are differences in detail between definitions of MS, above-threshold values of each component are typically scored as one point, with a total score of 3 or above indicative of MS. The construct of MS has succeeded in raising awareness among professionals and the public of cardiometabolic risk at the individual and population levels and focusing attention on correcting abdominal obesity and insulin resistance and continues to be the focus of extensive research.

WC is the anthropometric component of the MS definition, and some authors even consider above-cutpoint WC values to be mandatory for diagnosis of MS. WC conceptually is considered a measure of abdominal obesity, but statistically it differs little from the even more widely used body mass index (BMI), as the correlation between the two in large population studies is typically close to 0.9. Given this high correlation, BMI has even been taken to replace WC for the definition of MS in cohorts where WC data were unavailable [2]. A derivative of WC and hip circumference (HC) that is also taken to indicate abdominal obesity is WC/HC ratio [3–6], which has lesser, but still substantial correlation with BMI of ∼0.4 [7].

A line of research that responds to these concerns about the independence of anthropometric criteria has defined a body shape index (ABSI), based on a power-law expression that relates WC to height and weight (or equivalently height and BMI) [8]. Height (H), BMI, and ABSI then are complementary and independent indices that express the information provided by the basic measures of weight, height, and WC. Similarly, a hip index (HI) was defined that normalizes HC for BMI [7].

ABSI and HI, unlike BMI, show strong sex and age dependence. Accordingly, the risk attributable to ABSI and HI is best expressed after transforming the raw values, for example to *Z* scores adjusted for age and sex [7, 9]. Taking advantage of the approximate statistical independence of the indices H, BMI, ABSI, and HI, an anthropometric risk indicator (ARI) was introduced, which combines hazard ratios attributable to *Z* scores of the multiple independent indices and results in improved discrimination of mortality risk compared to using any one index in isolation [7, 10].

MS is known to be a risk factor for all-cause mortality, as well as onset of cardiovascular disease and diabetes [11], although whether it improves prediction over just using some of its individual components continues to be investigated [2, 12, 13]. Similarly, the anthropometric indices combined into ARI, particularly ABSI, have been shown to predict mortality hazard and morbidity onset [14–25].

BMI and ABSI have been shown to correlate with the components of MS [26–35]. However, there is limited information on how anthropometric risk (as measured by ARI) overlaps with metabolic risk (as measured by MS score) in populations.

In this study, we aim to answer two major questions. First, how do anthropometric indices based on H, W, WC, and HC correlate with MS in a general population sample? Second, how do anthropometry-based risk and MS complement each other as predictors of mortality hazard?

## 2. Methods

### 2.1. Data

The Third National Health and Nutrition Examination Survey (NHANES III) sampled the civilian noninstitutionalized USA population during 1988–1994 using a cluster approach, with some groups of public health interest (children, the elderly, black and Mexican-American people) deliberately oversampled [36]. Mortality outcomes for adult subjects were available from the National Center for Health Statistics with follow-up through 2011 (17–23 years of follow-up). We analyzed NHANES III public-use data for all nonpregnant adults (age 18 and over) with the required measurements and mortality follow-up. About half of the NHANES III cohort were examined in the morning, and hence have the fasting triglyceride and glucose measurements that are used to define MS.

### 2.2. ARI and MS

Anthropometric indices were calculated as follows [7, 8, 37]:

(1) $$\begin{array}{c} \text{BMI}\equiv \text{W}\cdot {\text{H}}^{-2},\\ \text{ABSI}\equiv \text{WC}\cdot {\text{H}}^{5/6}\cdot {\text{W}}^{-2/3},\\ \text{HI}\equiv \text{HC}\cdot {\left(\frac{\text{H}}{\langle \text{H}\rangle }\right)}^{0.310}\cdot {\left(\frac{\text{W}}{\langle \text{W}\rangle }\right)}^{-0.482}, \end{array}$$

where 〈H〉 = 166 cm and 〈W〉 = 73 kg are average values included in the definition as scaling factors.

The anthropometric index values for the NHANES III cohort are converted to *Z* scores by subtracting the age and sex specific mean and dividing by the standard deviation [7]. Penalized spline Cox proportional hazard regressions on the subset of NHANES III that did not have the data needed to determine MS score was used to estimate the natural logarithm of the mortality hazard as a function of each anthropometric index taken alone. Then, these estimated log hazard ratios (resp. designated here hH, hBMI, hABSI, and hHI) are computed for the *Z* score values of each subject in NHANES III and summed to give ARI. The estimated mortality hazard for each combination of anthropometric indices, as a fraction of the population mean hazard, is exp(ARI), so that ARI of 0 connotes population-average risk, while positive ARI connotes above-average risk and negative ARI below-average risk [7]. An online calculator that converts anthropometrics to *Z* scores and gives ARI and its components is available at https://www.nirkrakauer.net/sw/ari-calculator.html.

As previously done for NHANES III [38, 39], MS was defined following the Third Report of the National Cholesterol Education Program Adult Treatment Panel (ATP III) [40]. This relies on the presence or absence (scored 1/0) of each of five components: abdominal obesity (defined using WC), hypertension, hypertriglyceridemia, low high-density lipoprotein (HDL) levels, and hyperglycemia. The exact criteria used are given in Table 1. The summed score can range between 0 and 5, with a score of 3 or above constituting MS.

### 2.3. Prediction Models and Analysis

The considered mortality predictors in Cox proportional hazard modeling were the anthropometric index (H, BMI, ABSI, and HI) hazard ratio natural logarithms and their sum, the ARI; and the MS components (coded 0-1), MS score (0–5), and MS occurrence (0-1). The resulting predictive models considered are listed and explained in Table 2. In line with previous analyses of NHANES III [7], each prediction model also includes black race (coded 0-1) and sex. Age was implicitly included, being the timescale in the Cox model [8]. We used the provided sample weights for the morning subsample [36] so that our results would be better estimates for associations with mortality hazard in the general USA population.

As in [7], the main measure of relative predictive performance was AIC difference score, Δ_*i*_. For the best-performing model (with lowest AIC) Δ_*i*_=0, while other models have positive Δ_*i*_ [41]. Δ_*i*_ > 6 indicated models that perform significantly worse than the best-performing model (at the 95% confidence level) as mortality predictors for the sampled population [17]. We also calculated coefficients *R*^2^, denoting the proportion of variation in mortality explained by the predictors of each model, so that higher *R*^2^ suggests a model with greater explanatory power [42]. Another measure of model performance considered was concordance (*C*), defined as the fraction of pairs of individuals in the sample for which the one modeled to be at greater risk actually died sooner [43]. Concordance ranges from 0 to 1, with 0.5 the expected value for models with no skill and higher values indicating models that are more skillful at explaining variation in survival. To better understand the relationship between ARI, MS, and their components in the NHANES III population, we also show and discuss the correlation coefficients between them.

## 3. Results

There were 5221 nonpregnant adults in NHANES III who had recorded all the measurements needed to evaluate the anthropometric indices and MS components, out of whom 1564 (30%) died during follow-up. Based on the definition used, 1449 people had MS at baseline, for an MS prevalence of 28%. Mean ARI was near zero (−0.01), with a standard deviation of 0.23.

Correlation coefficients between the ARI-component hazard ratios based on H, BMI, ABSI, and HI are consistently very low in magnitude (under 0.1), which suggests that the anthropometric indices chosen do measure statistically independent components of mortality risk (Table 3). ARI is the sum of these components, and their respective correlations with ARI show that BMI is the largest contributor to anthropometric mortality risk in NHANES III, followed by ABSI and then HI, with H having negligible value for predicting mortality. The MS components, in turn, are intercorrelated, with correlation coefficients typically around 0.2, consistent with their being parts of a larger syndrome that nevertheless each add independent information (Table 3). They each have correlations of 0.5–0.7 with their sum, the MS score, and 0.4–0.6 with MS occurrence, with TG being the best-correlated single component (Table 3).

ARI correlates positively with each of the MS components, with the best correlation being with Waist. In fact, ARI correlates better with the MS components than any one ARI component does. ARI has a correlation of 0.36 with MS score and 0.27 with MS (Table 3). Anthropometric mortality risk is thus associated with MS, but not as closely associated with MS as any of the MS definition components are.

The fitted coefficients and performance indicators of the proportional hazard models for mortality prediction are given in Table 4. The presence of MS increased mortality hazard by 37% (95% confidence interval: 22–54%, model MS in Table 4). MS score was a better population predictor than MS occurrence, with each MS component present increasing mortality hazard by 15% (10–20%, model MS score). Separating the individual MS components suggests that Waist and TG are not significantly associated with mortality, while BP, HDL, and Glu are significantly and about equally associated with mortality (model MS components).

The predictive model with ARI (model ARI) significantly outperformed the models with only MS occurrence, MS score, or MS components. However, a model with both ARI and MS performed even better, with both ARI and MS remaining as significant predictors (model ARI + MS). ARI could also be combined with MS components, whereupon BP, HDL, and Glu remained significant mortality predictors (model ARI + MS components). In that case, the Waist component of MS was just as well omitted (models ARI + MSx score and ARI + MSx components), both because ARI already has WC as an input and because Waist's association with mortality risk in this cohort was not significant.

Overall, among the models considered here, ARI + MSx components, ARI + MS components, and ARI + MSx score were statistically tied for best predicting mortality hazard as measured by Δ_*i*_. While the MS score significantly outperformed MS occurrence as a mortality predictor, there was not a firm basis for giving different weights to the MS components to improve mortality prediction, as the performances of MS components and MS score and ARI + MSx components and ARI + MSx score, respectively, were statistically tied (Table 4).

## 4. Discussion

Here, we for the first time evaluated, for mortality prediction in the general population, the combination of a systematically selected set of independent anthropometrics (H, BMI, ABSI, and HI, whose respective attributable hazards were summed to give ARI) with MS. We found that although ARI was the best single mortality predictor and was positively correlated with all MS components, the clinical and laboratory data that contribute to the MS score could be used synergistically with ARI to further improve mortality prediction. Such individualized risk information could potentially be useful in a variety of clinical contexts for guiding personalized medical care [44–46].

There are some limitations to our study. Our cohort was relatively small in size, limiting statistical power, because many of the NHANES III sample did not have the fasting glucose and triglyceride measurements needed to evaluate MS. The long follow-up for mortality of approximately 20 years is a strength, but it is possible that associations of anthropometrics and MS components with mortality hazard have changed over time so that these findings would not be fully applicable to current patients. Possible time changes in associations with mortality have been most fully studied for BMI, with inconclusive results so far [47, 48]. As well, the ATP III definition of MS we used is one that has been employed in previous analyses of NHANES III [38, 39, 49], but many definitions have been proposed, with no single consensus [1, 50]. While most patients retain their MS classification across definitions [51], in some cases morbidity and mortality associations were affected by the definition used [13, 52].

We found that hypertension, hyperglycemia, and low HDL are the main drivers of the association of MS with mortality hazard in NHANES III. Some studies have found hyperglycemia to be the component of MS most associated with mortality [2, 13]. The strength of the mortality association with MS found in NHANES III is comparable to that seen in other cohorts [11].

It would be interesting to compare the risk associations seen with ARI and MS in the NHANES III national USA sample with those from other countries. WC and BMI cutoffs for cardiometabolic risks may vary between ethnicities [53, 54], although ABSI, for example, has been found to have comparable associations as a mortality predictor across cohorts from several continents [21–25, 55, 56]. ARI and MS could also be considered for prediction of morbidity such as cardiovascular disease and diabetes, for which the relative power of MS would be expected to be greater [11, 12].

Given the weak performance of a WC cutoff for determining mortality risk, replacing the Waist component in the definition of MS by a more sensitive anthropometric indicator of abdominal obesity could be worth exploring. Possible candidates are the WC/H ratio [15, 57–59], which is linked to body roundness in an elliptical model of the human body [60] and ARI itself (at least the sum of the BMI and ABSI risks, which show the largest correlations with WC). Similarly, given the lack of association of high TG with mortality, one could also consider elimination of the TG component in the definition of MS in favor of other measures such as elevated low-density lipoprotein cholesterol [61] or TG/HDL ratio [62]. Larger cohorts and a fuller range of health outcomes would probably be necessary to definitively show the merits of these proposals.

## 5. Conclusions

In summary, we found that anthropometric parameters and factors included in the definition of metabolic syndrome, while not uncorrelated, can function synergistically as predictors of mortality hazard, potentially improving individualized risk assessment compared to using either set of predictors in isolation.

## Figures and Tables

**Table 1:** Metabolic syndrome components.

Component	Criteria
Waist	Waist circumference above 102 cm for men or 88 cm for women.

BP	Systolic blood pressure at or above 130 mmHg or diastolic blood pressure at or above 85 mmHg or self-reported to be using blood pressure medications.

TG	Fasting serum triglycerides at or above 150 mg/dL.

HDL	Serum high-density lipoprotein cholesterol under 40 mg/dL for men, 50 mg/dL for women.

Glu	Fasting plasma glucose level at or above 110 mg/dL or self-reported to be taking pills for diabetes.

Abbreviations for and definitions of metabolic syndrome components used in this study. Each component is scored 1 if the criteria for it are met and 0 otherwise. A total score of 3 or above defines metabolic syndrome.

**Table 2:** Predictive models considered.

Model	Description
Base	No anthropometric or MS predictors.
ARI	ARI as a predictor.
MS	MS occurrence as a predictor.
MS score	MS score as a predictor.
ARI + MS	ARI and MS occurrence as predictors.
ARI + MS score	ARI and MS score as predictors.
ARI + MSx score	ARI and MS score (excluding Waist) as predictors. Excluding the WC component from the MS score was hypothesized to be reasonable when considering it alongside ARI since the BMI and ABSI components of ARI already correlate strongly with WC.
MS components	The 5 MS components as individual predictors. This tested the relative utility of the components of MS, which could suggest refinements of its definition for testing in future work.
ARI + MS components	ARI and the 5 MS components (Table 1) as individual predictors.
ARI + MSx components	ARI and the 4 MS components (excluding Waist) as individual predictors.

Models for predicting mortality that were compared in this study. MS: metabolic syndrome; ARI: anthropometric risk index; WC: waist circumference; BMI: body mass index; ABSI: a body shape index.

**Table 3:** Correlations in NHANES III.

	hH	hBMI	hABSI	hHI	ARI	Waist	BP	TG	HDL	Glu	MS score	MS
hH	1	−0.015	−0.012	−0.009	−0.015	0.074	0.030	−0.051	−0.014	−0.027	0.008	0.006
hBMI		1	0.032	0.080	0.775	0.388	0.116	0.104	0.123	0.100	0.284	0.215
hABSI			1	0.050	0.613	0.258	0.025	0.120	0.112	0.047	0.193	0.148
hHI				1	0.321	0.122	0.075	0.077	0.063	0.110	0.147	0.128
ARI					1	0.464	0.117	0.164	0.170	0.127	0.355	0.274
Waist						1	0.242	0.231	0.230	0.193	0.652	0.514
BP							1	0.202	0.033	0.231	0.584	0.438
TG								1	0.325	0.204	0.655	0.597
HDL									1	0.120	0.584	0.482
Glu										1	0.520	0.461
MS score											1	0.828
MS												1

Correlation coefficients among NHANES III nonpregnant adults. hH, hBMI, hABSI, and hHI refer to hazard ratio logarithms based on functional relationships between mortality and the Z scores of the respective anthropometric measures height, body mass index, a body shape index, and hip index. ARI is the sum of these 4 quantities. The metabolic syndrome score MS score is the sum of scores for the waist circumference, blood pressure, triglyceride, high-density lipoprotein, and glucose components defined in Table 1. Metabolic syndrome MS is defined as a score of 3 or more.

**Table 4:** Mortality hazard association with body measures and metabolic syndrome in NHANES III.

Model	Δ_*i*_	*R* ^2^	*C*	Predictor	HR
Base	97.3	0.0148	0.543		

ARI	27.1	0.0426	0.600	ARI	2.93 (2.30–3.73)

MS	71.2	0.0257	0.563	MS	1.37 (1.22–1.54)

MS score	55.1	0.0318	0.582	MS score	1.15 (1.10–1.20)

ARI + MS	19.1	0.0464	0.604	ARI	2.65 (2.06–3.40)
				MS	1.22 (1.08–1.37)

ARI + MS score	10.2	0.0498	0.610	ARI	2.52 (1.95–3.25)
				MS score	1.10 (1.05–1.15)

ARI + MSx score	5.5	0.0516	0.610	ARI	2.62 (2.05–3.36)
				MSx score	1.13 (1.08–1.19)

MS components	49.9	0.0369	0.598	Waist	1.12 (0.99–1.27)
				BP	1.27 (1.10–1.45)
				TG	0.94 (0.82–1.07)
				HDL	1.23 (1.08–1.40)
				Glu	1.31 (1.15–1.49)

ARI + MS components	1.3	0.0562	0.617	ARI	2.68 (2.07–3.49)
				Waist	0.95 (0.83–1.08)
				BP	1.26 (1.10–1.44)
				TG	0.93 (0.81–1.06)
				HDL	1.23 (1.08–1.40)
				Glu	1.25 (1.09–1.43)

ARI + MSx components	0	0.0560	0.620	ARI	2.59 (2.02–3.33)
				BP	1.25 (1.09–1.44)
				TG	0.92 (0.81–1.05)
				HDL	1.22 (1.07–1.38)
				Glu	1.24 (1.09–1.42)

Results of Cox proportional hazard modeling for mortality risk in NHANES III with the linear predictors shown. All models also included as predictors sex and race. The hazard ratios are given with 95% confidence intervals. Δ_*i*_ = Akaike information criterion score difference relative to the best-performing model shown (see Methods for details); *R*^2^ = measure of explained variation; *C* = concordance; HR = hazard ratio.

## Data Availability

The NHANES III data used to support the findings of this study are publicly available online from the United States National Center for Health Statistics
